# 

**Published:** 1909-07-17

**Authors:** 


### BOOKS RECEIVED.

Cassell and Company, Limited.
"The Science and Art of Nursing." Vol. IV. By Medical and Nursing Authorities.

Eyre and Spottiswoode.
"English Church Pageant Handbook."

Sir Isaac Pitman and Sons, Ltd.
"Body and Soul." By Percy Dearmer, M.A.

Edward Arnold.
"The Sanitary Officer's Handbook of Practical Hygiene." By Major C. F. Wanhill, R.A.M.C., and Major W. W. D. Beveridge, R.A.M.C.

J. and A. Churchill.
"An Atlas of Dental Extractions," by C.E. Wallis.

John Bale, Sons and Danielsson, Ltd.
"The Medico-Chirurgical Series—I." "The Practice of Anæsthetics." By R. W. Collum, L.R.C.P., etc. "General Surgical Technique." By H. M. W. Gray, M.B., C.M., etc. "Synoptic Chart of Cardiac Examination."
"The Campaign Against Microbes." By Etienne Burnet, M.D.

THE

### GRADUATES' MEDICAL SCHOOLS.

DIARY FOR THE WEEK, JULY 19 to 24.

### THE HOSPITAL FOR SICK CHILDREN, Great Ormond Street, W.C.

**At 4 p.m.**
**July 22,** Dr. Thursfield, **Whooping Cough.**

The fees for attending the lectures and demonstrations at the Hospital for Sick Children have been revised as follows:—Clinical clerks, special fee for three months, £3 3*s.*; Students' Ticket, three months, £5 5*s.*; Perpetual Ticket, £10 10*s.*

### CENTRAL LONDON THROAT AND EAR HOSPITAL, Gray's Inn Road, W.C.

**At 3.45 p.m.**
**July 20,** Dr. W. H. George, **Anæsthetics.**
**July 23,** Mr. W. Wallis, **Mouth and Teeth.**

### MEDICAL GRADUATES' COLLEGE AND POLYCLINIC, 22 Chenies Street, W.C.

**At 4 p.m.**
**July 19,** Dr. J. Galloway, **Skin.**
**July 20,** Dr. Wm. Ewart, **Medical.**
**Juty 21,** Mr. Cecil H. Leaf, **Surgical.**
**July 22,** Sir Jonathan Hutchinson, **Surgical.**
**At 5.15 p.m.**
**July 19,** Dr. G. C. Cathcart, **Stammering.**
**July 20,** Dr. Leonard Guthrie, **Some Nervous Affections in Children.**
**July 21,** Dr. E. I. Spriggs, **The Prescription of Dietaries.**
**July 22,** Mr. Arthur Edmunds, **Chemical Antiseptics in Modern Surgery.**
**July 23,** *Collrge closes for Vacation.*

